# Development and Validation of a Machine Learning–Based Model for Predicting All-Cause Mortality Risk in Patients With Type 2 Diabetes Mellitus Combined With Hypertension: National Cohort Study

**DOI:** 10.2196/85557

**Published:** 2026-09-22

**Authors:** Wenlong Ding, Lei Fang, Cunming Fang

**Affiliations:** 1 Xuancheng Hospital Affiliated to Wannan Medical College (Xuancheng People's Hospital) Xuancheng China; 2 The People's Hospital Tongling Tongling China

**Keywords:** type 2 diabetes mellitus, hypertension, machine learning, mortality prediction, risk stratification, National Health and Nutrition Examination Survey, NHANES, artificial intelligence, AI

## Abstract

**Background:**

Type 2 diabetes mellitus (T2DM) combined with hypertension significantly increases mortality risk, yet accurate risk prediction models remain limited.

**Objective:**

We aimed to develop and validate machine learning–based models to predict all-cause mortality in patients with T2DM and hypertension.

**Methods:**

We analyzed data from the National Health and Nutrition Examination Survey from 1999 to 2018 linked with mortality data up to December 31, 2019. Adult participants (aged ≥20 years) with concurrent T2DM and hypertension were included. Five machine learning algorithms were developed and compared: random forest, light gradient boosting machine, decision tree, extreme gradient boosting, and logistic regression. Model performance was evaluated using area under the curve (AUC), calibration plots, and decision curve analysis.

**Results:**

A total of 2428 participants were included (mean age 62.05, SE 0.33 years; n=1218, 50.15% female). During a median follow-up of 6.75 (IQR 4.20-8.90) years, among the 2428 patients, 719 (29.6%) deaths occurred. The random forest model demonstrated superior performance (AUC=0.873, 95% CI 0.856-0.891) compared to light gradient boosting machine (AUC=0.785), decision tree (AUC=0.732), extreme gradient boosting (AUC=0.792), and logistic regression (AUC=0.783). Key predictive features included age, race, chronic kidney disease, BMI, and blood urea nitrogen. The model exhibited excellent calibration and clinical utility across various risk thresholds.

**Conclusions:**

Our machine learning–based model provides accurate all-cause mortality prediction for patients with T2DM and hypertension, potentially supporting clinical decision-making and risk stratification in this high-risk population.

## Introduction

Type 2 diabetes mellitus (T2DM) and hypertension represent the most prevalent chronic noncommunicable diseases worldwide, with their coexistence being remarkably common. Recent epidemiological data indicate that approximately 75% of patients with T2DM concurrently have hypertension [1]. This comorbid state creates a synergistic pathophysiological environment that significantly amplifies the risk of cardiovascular events, kidney disease progression, and all-cause mortality, with patients with T2DM and hypertension facing a 2- to 4-fold increased risk of all-cause mortality compared to healthy populations [2,3]. A recent meta-analysis encompassing 1 million patients confirmed that this population experiences a 3.2-fold increased risk of cardiovascular death and a 2.8-fold increased risk of all-cause mortality [4].

Traditional risk assessment tools demonstrate limited predictive accuracy in patients with T2DM and hypertension, typically achieving C-statistics below 0.75 [5,6]. These conventional scoring systems primarily rely on traditional statistical methods such as Cox regression or logistic regression (LR), which have inherent limitations in handling complex nonlinear relationships and high-dimensional variable interactions, failing to fully exploit the predictive potential of modern clinical data [7].

Machine learning (ML) technologies provide new opportunities to address these challenges. Ensemble learning algorithms such as gradient boosting decision tree (DT) and random forest (RF) can automatically identify nonlinear patterns and higher-order interactions, demonstrating superior performance compared to traditional statistical models [8,9]. Weng et al [10] confirmed that ML algorithms achieved area under the curve (AUC) improvements of 0.074 over traditional models in cardiovascular risk prediction using UK primary care databases. However, the application of ML for all-cause mortality prediction in patients with diabetes and hypertension remains limited, with existing studies predominantly confined to small samples or specific end points, lacking systematic development based on large-scale, nationally representative populations [11].

From a clinical perspective, management of patients with T2DM and hypertension requires individualized risk stratification. High-risk patients necessitate aggressive interventions and intensive monitoring, whereas low-risk patients may be suitable for routine management strategies [12]. Accurate risk prediction models assist clinicians in developing individualized treatment plans and optimize health care resource allocation [13,14]. ML models combined with interpretability techniques such as Shapley additive explanations (SHAP) analysis provide high-precision predictions while elucidating individual feature contributions, offering transparent decision support tools [15,16].

The rapid growth in patients with T2DM and hypertension presents enormous health care challenges. The International Diabetes Federation predicts that the number of global patients with diabetes will reach 780 million by 2045, with approximately 60% having concurrent hypertension [17]. ML-based precision risk prediction can help health care systems identify high-risk populations for optimal resource allocation under resource-limited conditions [18].

While ML approaches for mortality prediction have been increasingly explored, existing studies in patients with T2DM and hypertension face several critical limitations that our study specifically addresses. First, most prior ML studies in populations with diabetes focus on cardiovascular end points or diabetes complications rather than all-cause mortality, which is the most comprehensive outcome measure for this high-risk population. Second, existing studies predominantly use small, single-center cohorts or specific health care system databases, limiting generalizability to broader populations. Third, few studies have systematically compared multiple state-of-the-art algorithms using rigorous feature selection methods specifically optimized for this comorbid population.

Therefore, developing an ML-based all-cause mortality risk prediction model specifically for patients with T2DM and hypertension represents both significant clinical value and an urgent need in precision medicine. This study leveraged nationally representative National Health and Nutrition Examination Survey (NHANES) data to construct an accurate, interpretable, and generalizable risk prediction tool for improving clinical outcomes in this high-risk population.

## Methods

### Study Population and Data Sources

This study used data from the NHANES, a continuous cross-sectional survey conducted by the National Center for Health Statistics designed to assess the health and nutritional status of the US population. We included data from NHANES cycles spanning 1999 to 2018 linked with mortality data from the National Death Index up to December 31, 2019. Adult participants (≥20 years) with concurrent T2DM and hypertension were eligible for inclusion. T2DM was defined as fasting glucose of 7.0 mmol/L or higher or 2-hour oral glucose tolerance test results of 11.1 mmol/L or higher, random blood glucose of 11.1 mmol/L or higher, and hemoglobin A_1c_ of 6.5% or higher. Additionally, participants using diabetes medications or insulin or those with physician-confirmed diabetes diagnoses were also considered eligible [19]. Hypertension was defined as (1) self-reported physician diagnosis, (2) current use of antihypertensive medications, and (3) average systolic blood pressure of 140 mm Hg or higher or diastolic blood pressure of 90 mm Hg or higher from multiple measurements [20]. Exclusion criteria were (1) patients without T2DM, (2) patients without hypertension, (3) missing mortality follow-up data, and (4) patients with missing data for other covariates. Ultimately, a total of 2428 eligible participants were included in the analysis, with the study flowchart shown in Figure 1.

**Figure 1:**
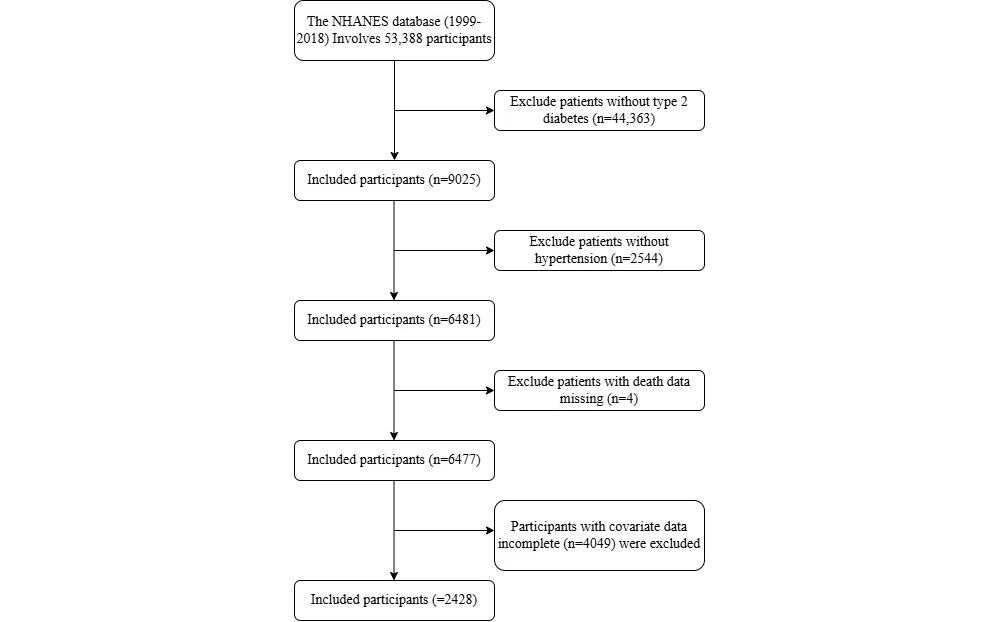
Study flowchart. NHANES: National Health and Nutrition Examination Survey.

### Death-Related Information

The primary outcomes of this study were all-cause mortality and cardiovascular disease mortality. To determine patient vital status, we linked mortality information of NHANES participants from 1999 to 2018 with death records from the National Death Index using probabilistic matching algorithms. Additionally, to estimate cardiovascular mortality, we applied *International Classification of Diseases, 10th Revision* code ranges, including I00 to I09, I11, I13, I20 to I51, and I60 to I69. For the follow-up period in this study, the end point was defined as patient death or December 31, 2019, whichever came first.

### Covariates

To control for confounding effects, multiple demographic and clinical characteristics were included. Demographic characteristics encompassed age, sex, race and ethnicity, educational level, poverty-to-income ratio (PIR), and marital status. Lifestyle factors included smoking status (never smoker, former smoker, and current smoker) and alcohol consumption (never drinker, former drinker, mild drinking, moderate drinking, and heavy drinking). Anthropometric measures included BMI. Medical history comprised hypertension, chronic kidney disease (CKD), chronic obstructive pulmonary disease (COPD), atherosclerotic cardiovascular disease (ASCVD), and hyperlipidemia. Medication use included antihypertensive agents, antidiabetic medications, and lipid-lowering drugs. Laboratory examinations encompassed complete blood count parameters (neutrophils, lymphocytes, monocytes, and hemoglobin), liver and kidney function markers (serum creatinine, uric acid, blood urea nitrogen [BUN], albumin, alanine aminotransferase, and aspartate aminotransferase), lipid profile (total cholesterol, high-density lipoprotein, low-density lipoprotein, and triglycerides), and glucose metabolism markers (hemoglobin A_1c_).

### Construction of ML Models

In this study, least absolute shrinkage and selection operator (LASSO) regression was used for variable selection due to its ability to simultaneously perform variable selection and model parameter estimation, effectively reduce model overfitting, improve model generalization capability, and provide strong interpretability for identifying key feature variables. The variables selected through LASSO regression were subsequently used for ML modeling and validation. We randomly divided the dataset into training and testing sets at a 7:3 ratio. The training set was used for computing and training model parameters, whereas the testing set was used to evaluate model performance. To address class imbalance issues, we applied the synthetic minority oversampling technique exclusively to the training set after the initial data split to generate synthetic samples of the minority class, thereby optimizing model performance [21]. Importantly, the synthetic minority oversampling technique was never applied to the testing set, ensuring that the test data remained completely independent and composed only of original samples, thus preventing any data leakage that could artificially inflate model performance. Five algorithms were selected (LR, light gradient boosting machine [LightGBM], extreme gradient boosting [XGBoost], DT, and RF) to construct ML models for predicting patient all-cause mortality risk. Additionally, to prevent overfitting and enhance model generalization capability, we used 10-fold cross-validation for evaluation and constructed the final models after repeated iterations. The optimal hyperparameters for all models can be found in Table S1 in Multimedia Appendix 1. Through grid search, we traversed all parameter combinations to identify the parameters yielding optimal overall performance. Model performance was evaluated using metrics including accuracy, AUC, precision, recall, and *F*_1_-score.

### Explainability of ML Models

The SHAP algorithm was used to assign corresponding attribute values (SHAP values) to each variable, ultimately yielding the model with optimal performance. SHAP is a game theory–based ML model prediction method. SHAP represents a unified framework for explaining ML predictions and serves as a novel approach for interpreting various black-box ML models. Each feature in every sample has a corresponding SHAP value. Positive SHAP values indicate that a feature has a positive influence on the prediction probability, whereas negative SHAP values suggest that a feature has a negative influence on the prediction probability. Therefore, the weighted average of SHAP values for each feature demonstrates the overall impact of each feature on the prediction model. Feature importance plots and summary beeswarm plots were used to reveal the relationships between features and the prediction model.

### Statistical Analysis

Data analysis was performed using the R software (version 4.3.2; R Foundation for Statistical Computing). Throughout the analysis process, we adhered to NHANES analysis and reporting guidelines, accounting for the complex sampling design and sampling weights. In weighted analyses, sample weights were applied for data processing. Continuous variables were expressed as means and SEs, whereas categorical variables were presented as frequencies and percentages. One-way ANOVA and Pearson chi-square tests were used for baseline characteristic comparisons to examine differences in continuous and categorical variables, respectively. Results were considered statistically significant when *P*<.05 (2 sided).

### Ethical Considerations

The NHANES protocol has been approved by the National Center for Health Statistics Ethics Review Board, and this study does not contain any personally identifiable information. Therefore, the ethics committee of Xuancheng People’s Hospital exempted this study from ethics approval requirements.

## Results

### Basic Characteristics of the Study Population

This study included patients with T2DM and hypertension, who were categorized into survival and death groups based on all-cause mortality during the follow-up period. Patients in the death group were significantly older (mean age 68.79, SE 0.58 vs 59.48, SE 0.37 years; *P*<.001). Racial distribution showed significant differences (*P*<.001), with a higher proportion of non-Hispanic White individuals in the death group (543/719, 75.69% vs 1114/1709, 65.22%). Marital status and educational level also exhibited significant differences, with higher proportions of unmarried individuals (207/719, 29.81% vs 265/1709, 15.49%) and those with lower educational attainment (232/719, 33.26% vs 318/1709, 18.62%) in the death group (*P*<.001 in all cases). Regarding lifestyle factors, the death group had higher proportions of former smokers (314/719, 44.45% vs 579/1709, 33.86%) and former drinkers (267/719, 38% vs 430/1709, 25.18%; *P*<.001 in all cases). The comorbidity burden was significantly higher in the death group, with notably higher prevalence rates of ASCVD (248/719, 34.46% vs 336/1709, 19.66%) and CKD (436/719, 61.66% vs 549/1709, 32.53%; *P*<.001 in all cases). COPD prevalence was also higher in this group (82/719, 11.81% vs 138/1709, 8.05%; *P*=.02). Laboratory parameters revealed more severe renal impairment in the death group, with significantly elevated serum creatinine (mean 100.86, SE 3.21 vs 83.03, SE 1.29 μmol/L) and BUN (mean 6.77, SE 0.17 vs 5.65, SE 0.07 mmol/L; *P*<.001) levels. Complete blood count analysis showed lower lymphocyte counts (mean 1.88, SE 0.04 vs 2.06, SE 0.02; *P*=.001). The survival group exhibited higher BMI (mean 34.08, SE 0.26 vs 31.19, SE 0.40 kg/m^2^) and higher PIR (mean 2.91, SE 0.06 vs 2.33, SE 0.08), indicating better socioeconomic status (*P*<.001 in all cases; Table 1).

**Table 1:** Basic characteristics of the study population (N=2428).

Variables				Total		Survival group (n=1709)		All-cause mortality group (n=719)		*P* value	
**Demographics**											
	Age (y), mean (SE)		62.05 (0.33)		59.48 (0.37)		68.79 (0.58)		<.001		
	**Sex, n (%); SE**										.54
		Female	1218 (50.15); 0.02		866 (50.65); 1.76		352 (48.83); 2.24				
		Male	1210 (49.85); 0.03		843 (49.35); 1.76		367 (51.17); 2.24				
	**Race and ethnicity, n (%); SE**										<.001
		Mexican American	161 (6.65); 0.01		130 (7.57); 0.84		31 (4.21); 0.87				
		Non-Hispanic Black	348 (14.35); 0.01		252 (14.75); 1.23		96 (13.29); 1.47				
		Non-Hispanic White	1657 (68.10); 0.04		1114 (65.22); 1.89		543 (75.69); 2.21				
		Other	262 (10.90); 0.01		213 (12.46); 1.16		49 (6.81); 1.21				
	**Marital status, n (%); SE**										<.001
		Divorced	318 (13.13); 0.01		228 (13.35); 1.22		90 (12.56); 1.51				
		Married	1456 (59.98); 0.03		1079 (63.14); 1.57		377 (51.66); 2.30				
		Never married	182 (7.46); 0.01		137 (8.03); 0.77		45 (5.97); 1.31				
		Other	472 (19.43); 0.01		265 (15.49); 0.99		207 (29.81); 1.95				
	**Educational level, n (%); SE**										<.001
		High school or equivalent	694 (28.58); 0.02		482 (28.17); 1.73		212 (29.64); 2.01				
		Lower than high school	550 (22.65); 0.01		318 (18.62); 1.35		232 (33.26); 2.33				
		Some college or higher	1184 (48.77); 0.02		909 (53.21); 1.85		275 (37.10); 2.42				
	PIR^a^, mean (SE)		2.75 (0.05)		2.91 (0.06)		2.33 (0.08)		<.001		
**Clinical measurements, mean (SE)**											
	BMI (kg/m^2^)		33.28 (0.22)		34.08 (0.26)		31.19 (0.40)		<.001		
	HbA_1c_^b^		6.90 (0.04)		6.92 (0.05)		6.85 (0.08)		.43		
**Major comorbidities, n (%); SE**											
	**ASCVD^c^**										<.001
		No	1844 (76.26); 0.03		1373 (80.34); 1.23		471 (65.54); 2.34				
		Yes	584 (23.74); 0.02		336 (19.66); 1.23		248 (34.46); 2.34				
	**CKD^d^**										<.001
		No	1443 (59.45); 0.03		1160 (67.47); 1.35		283 (38.34); 2.88				
		Yes	985 (40.55); 0.02		549 (32.53); 1.35		436 (61.66); 2.88				
	**COPD^e^**										.02
		No	2208 (90.92); 0.04		1571 (91.95); 0.89		637 (88.19); 1.44				
		Yes	220 (9.08); 0.01		138 (8.05); 0.89		82 (11.81); 1.44				
	**Hyperlipidemia**										.44
		No	256 (10.53); 0.01		173 (10.15); 0.89		83 (11.53); 1.52				
		Yes	2172 (89.47); 0.04		1536 (89.85); 0.89		636 (88.47); 1.52				
**Lifestyle factors, n (%); SE**											
	**Smoking status**										<.001
		Former	893 (36.78); 0.02		579 (33.86); 1.63		314 (44.45); 2.50				
		Never	1171 (48.22); 0.02		883 (51.68); 1.72		288 (39.09); 2.63				
		Currently	364 (15.01); 0.01		247 (14.45); 1.23		117 (16.46); 1.88				
	**Alcohol consumption**										<.001
		Former	697 (28.71); 0.02		430 (25.18); 1.55		267 (38.00); 2.49				
		Heavy	253 (10.43); 0.01		197 (11.55); 1.18		56 (7.49); 1.25				
		Mild	848 (34.91); 0.02		646 (37.80); 1.87		202 (27.30); 2.31				
		Moderate	256 (10.53); 0.01		189 (11.04); 1.19		67 (9.22); 1.63				
		Never	374 (15.42); 0.01		247 (14.44); 1.41		127 (17.99); 1.81				
**Renal function, mean (SE)**											
	Creatinine (μmol/L)		87.94 (1.43)		83.03 (1.29)		100.86 (3.21)		<.001		
	UA^f^		358.69 (2.77)		354.93 (3.22)		368.60 (4.92)		.02		
	BUN^g^ (mmol/L)		5.95 (0.07)		5.65 (0.07)		6.77 (0.17)		<.001		
**Liver function, mean (SE)**											
	ALT^h^		27.34 (0.48)		28.09 (0.55)		25.38 (0.99)		.02		
	AST^i^		26.36 (0.41)		26.13 (0.45)		26.96 (0.81)		.35		
	Albumin		4.13 (0.01)		4.13 (0.01)		4.10 (0.02)		.11		
**Complete blood count, mean (SE)**											
	Lymphocytes		2.01 (0.02)		2.06 (0.02)		1.88 (0.04)		.001		
	Monocytes		0.58 (0.01)		0.57 (0.01)		0.60 (0.01)		.03		
	Neutrophils		4.56 (0.05)		4.52 (0.05)		4.68 (0.08)		.08		
	Hemoglobin		14.16 (0.05)		14.21 (0.05)		14.03 (0.08)		.05		
**Lipid profile, mean (SE)**											
	TG^j^		1.70 (0.03)		1.68 (0.04)		1.78 (0.04)		.05		
	TC^k^		4.76 (0.03)		4.72 (0.04)		4.88 (0.06)		.01		
	HDL^l^		1.27 (0.01)		1.25 (0.01)		1.33 (0.04)		.03		
	LDL^m^		2.71 (0.03)		2.70 (0.03)		2.73 (0.05)		.61		
**Medication use, n (%); SE**											
	**Antidiabetics**										.02
		No	1044 (42.99); 0.02		705 (41.23); 1.68		339 (47.63); 2.22				
		Yes	1384 (57.01); 0.03		1004 (58.77); 1.68		380 (52.37); 2.22				
	**Antihypertensives**										.70
		No	440 (18.14); 0.01		314 (18.39); 1.35		126 (17.49); 1.95				
		Yes	1988 (81.86); 0.03		1395 (81.61); 1.35		593 (82.51); 1.95				
	**Antihyperlipidemics**										.02
		No	1149 (47.33); 0.02		778 (45.54); 1.61		371 (52.06); 2.24				
		Yes	1279 (52.67); 0.02		931 (54.46); 1.61		348 (47.94); 2.24				

^a^PIR: poverty-to-income ratio.

^b^HbA_1c_: hemoglobin A_1c_.

^c^ASCVD: atherosclerotic cardiovascular disease.

^d^CKD: chronic kidney disease.

^e^COPD: chronic obstructive pulmonary disease.

^f^UA: uric acid.

^g^BUN: blood urea nitrogen.

^h^ALT: alanine aminotransferase.

^i^AST: aspartate aminotransferase.

^j^TG: triglycerides.

^k^TC: total cholesterol.

^l^HDL: high-density lipoprotein.

^m^LDL: low-density lipoprotein.

### Important Factors for Screening the Risk of All-Cause Mortality in Patients With T2DM and Hypertension by LASSO Profile

To select the most suitable variables, LASSO regression was applied. As shown in Figure 2A, with an increase in log(λ), the coefficients in the model gradually approach 0, indicating that the independent variables are eliminated one by one. In Figure 2B, it can be observed that all variables are excluded at “lambda.1se,” leaving no coefficients in the model. On the basis of 10-fold cross-validation, LASSO ultimately identified age, sex, race, marital status, smoking status, alcohol consumption, ASCVD, CKD, COPD, antihyperlipidemic drug therapy, BMI, PIR, creatinine, BUN, lymphocyte count, monocyte count, and hemoglobin as the optimal independent variables in the training set.

**Figure 2:**
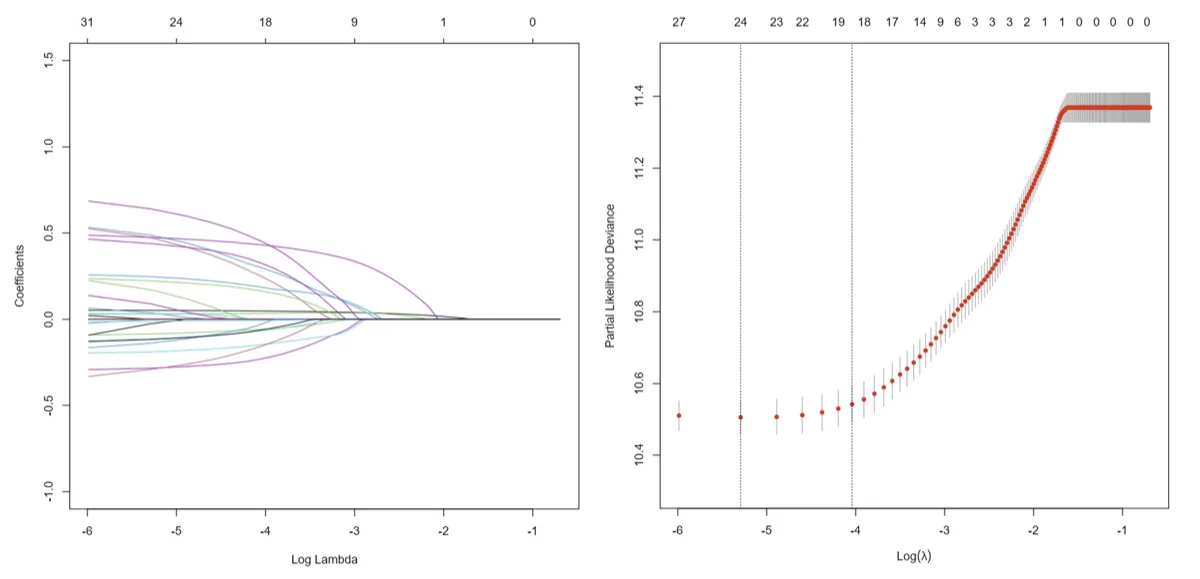
Presentation of the results of the least absolute shrinkage and selection operator (LASSO) regression analysis. (A) LASSO regression model screening variable trajectories. (B) LASSO regression model factor selection: the left dashed line represents the optimal λ value (“lambda.min”), whereas the right dashed line marks the λ value within 1 SE of the optimal (“lambda.1se”).

### Model Development and Validation

In this study, LR, LightGBM, XGBoost, RF, and DT were used to construct ML models, with 70% of the data used for training and 30% used for testing. Figures 3 and 4 show the receiver operating characteristic curves of the 5 ML models on both the training and validation sets. The RF model’s receiver operating characteristic curve was closest to the upper left corner on the training set, indicating superior performance. Figures 5 and 6 show the accuracy, AUC, precision, recall, and *F*_1_-score metrics. As shown in Figures 5 and 6, the RF algorithm exhibited optimal performance in the training set and achieved acceptable accuracy in the testing set. Decision curve analysis and calibration curves in Figures S1 and S2 in Multimedia Appendix 1 further confirmed the clinical utility of the RF model. Therefore, RF was selected for subsequent analysis.

**Figure 3:**
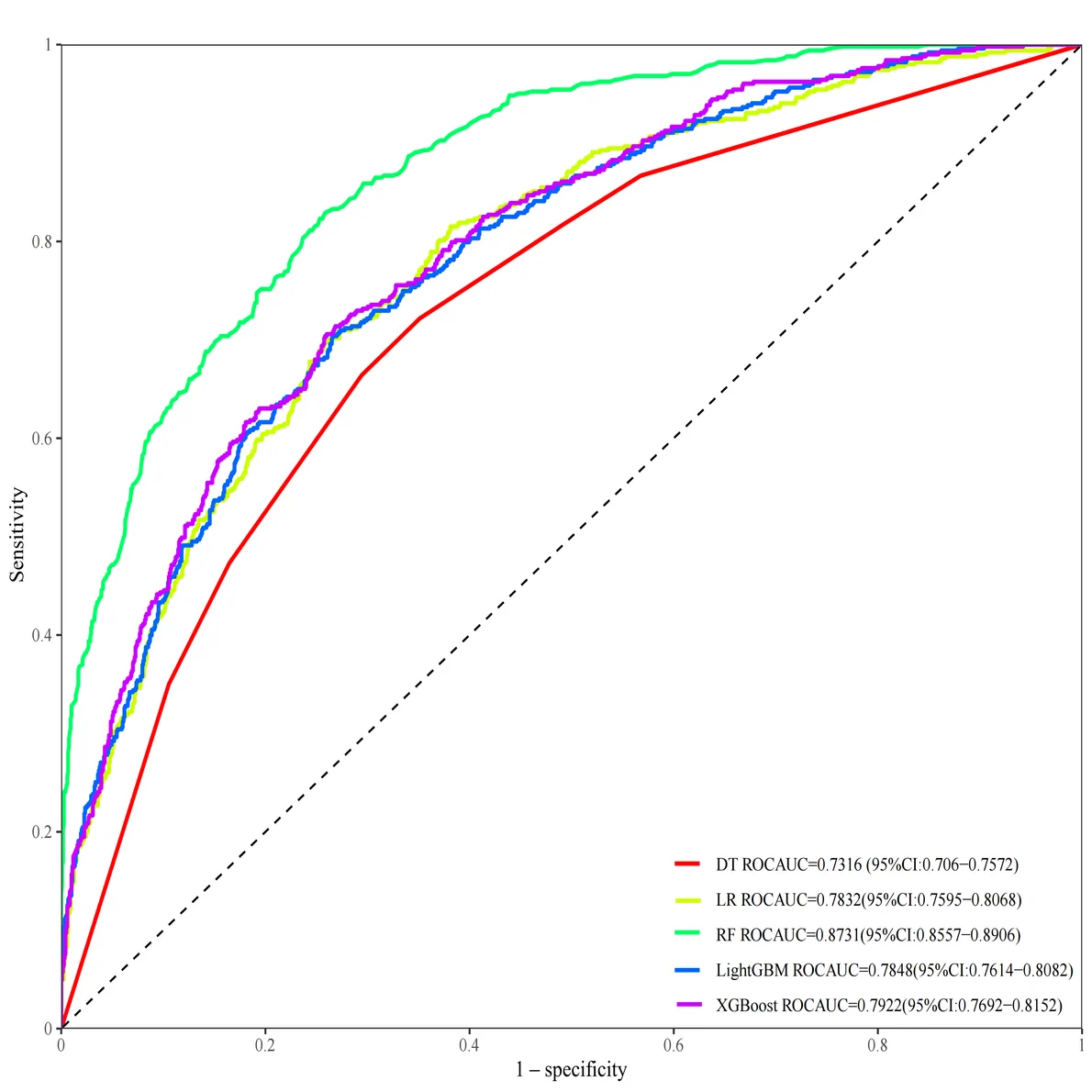
Receiver operating characteristic curve analysis of the machine learning models training set. DT: decision tree; LightGBM: light gradient boosting machine; LR: logistic regression; RF: random forest; ROCAUC: area under the receiver operating characteristic curve; XGBoost: extreme gradient boosting.

**Figure 4:**
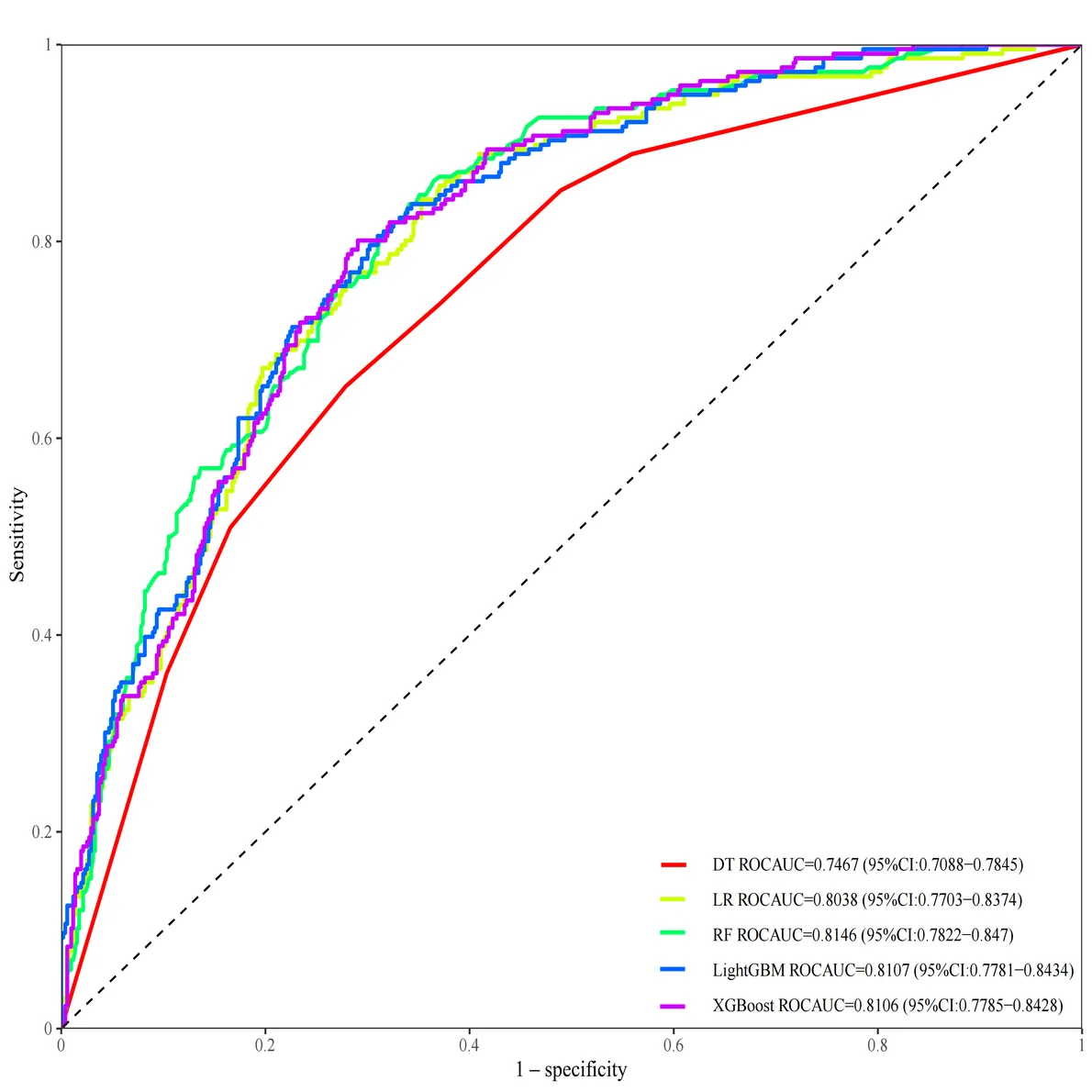
Receiver operating characteristic curve analysis of the machine learning models validation set. DT: decision tree; LightGBM: light gradient boosting machine; LR: logistic regression; RF: random forest; ROCAUC: area under the receiver operating characteristic curve; XGBoost: extreme gradient boosting.

**Figure 5:**
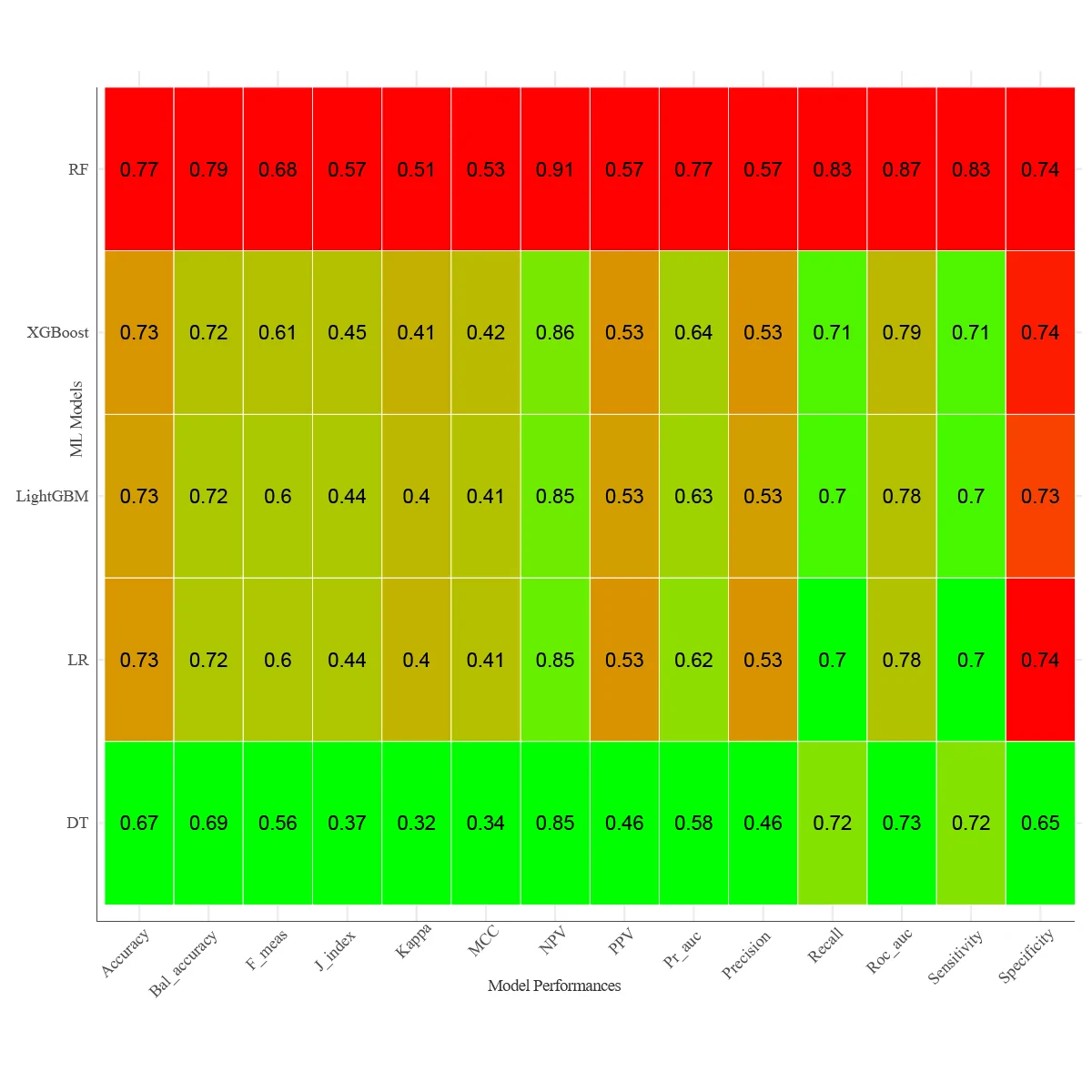
Performance comparison of different machine learning models on the training sets across multiple evaluation metrics. The heat maps display the performance of decision tree (DT), extreme gradient boosting (XGBoost), logistic regression (LR), random forest (RF), and light gradient boosting machine (LightGBM). Each cell represents the value of a specific evaluation metric: accuracy, balanced accuracy (Bal_accuracy), F1-score (F_meas), J-index, κ, Matthew correlation coefficient (MCC), positive predictive value (PPV), negative predictive value (NPV), precision, recall, area under the receiver operating characteristic curve (ROC_AUC), sensitivity, and specificity. Higher values are indicated in red, whereas lower values are indicated in green, showing the model’s effectiveness in both the training and test sets. PR_AUC: area under the precision-recall curve.

**Figure 6:**
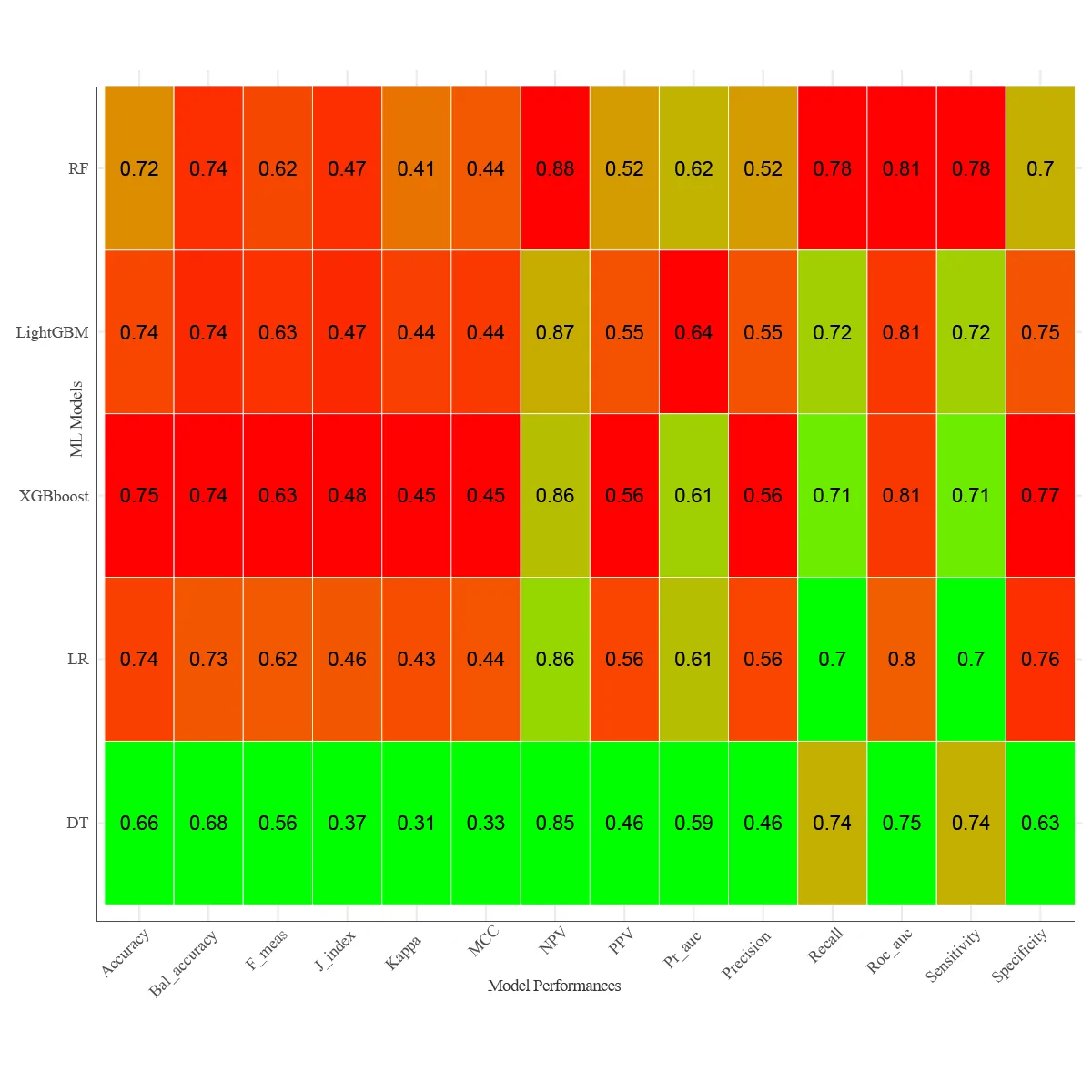
Performance comparison of different machine learning models on the test sets across multiple evaluation metrics. The heat maps display the performance of decision tree (DT), extreme gradient boosting (XGBoost), logistic regression (LR), random forest (RF), and light gradient boosting machine (LightGBM). Each cell represents the value of a specific evaluation metric: accuracy, balanced accuracy (Bal_accuracy), F1-score (F_meas), J-index, κ, Matthew correlation coefficient (MCC), positive predictive value (PPV), negative predictive value (NPV), precision, recall, area under the receiver operating characteristic curve (ROC_AUC), sensitivity, and specificity. Higher values are indicated in red, whereas lower values are indicated in green, showing the model’s effectiveness in both the training and test sets. PR_AUC: area under the precision-recall curve.

### Model Interpretation

We conducted SHAP analysis on the RF model to evaluate the importance of each feature and its impact on model prediction outcomes (Figures 7 and 8). The results demonstrated that age was the most critical variable, exhibiting the highest SHAP value and playing a dominant role in predicting all-cause mortality risk in patients with T2DM and hypertension. Additionally, factors such as race, CKD, BMI, and BUN also exerted influences on patient risk.

**Figure 7:**
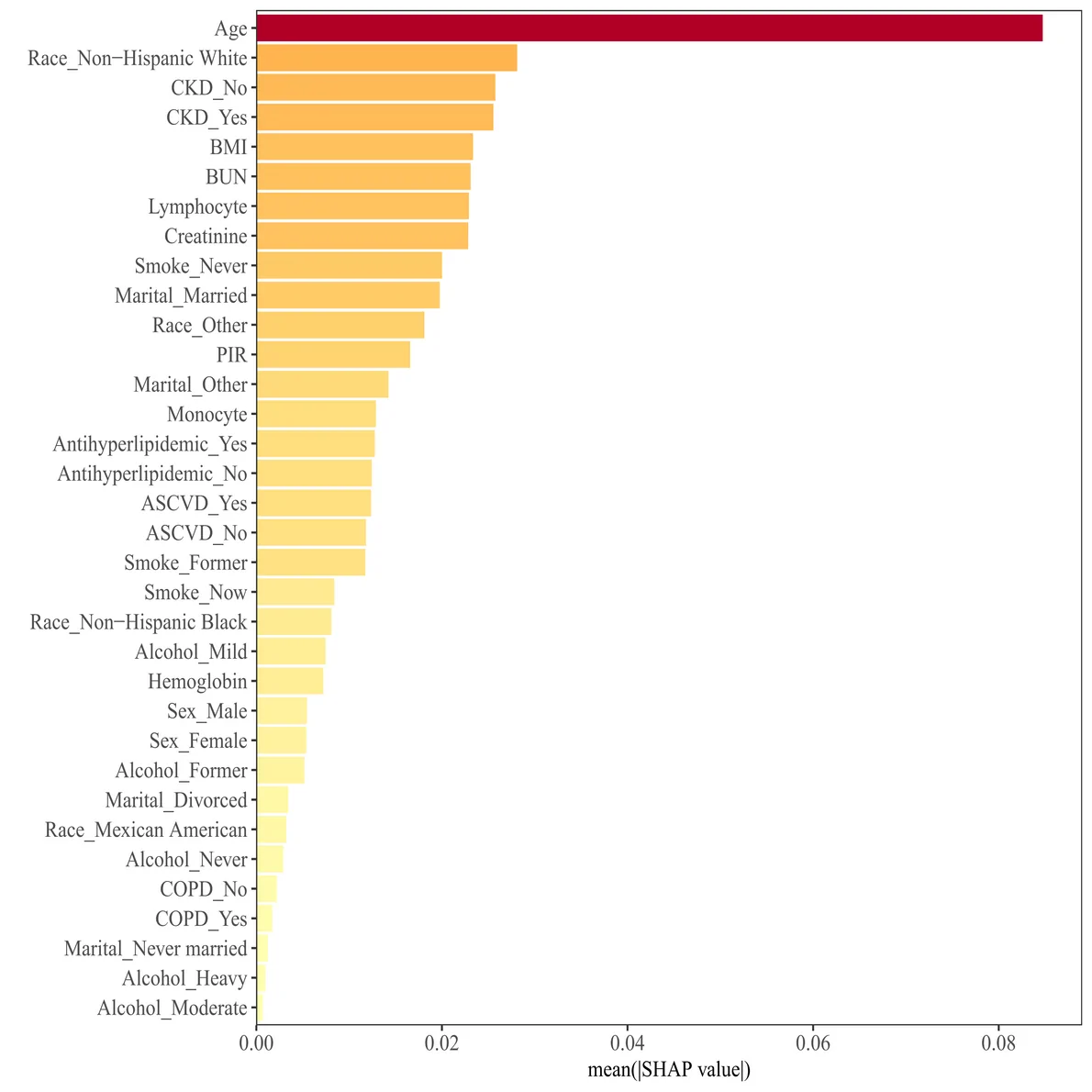
Feature-ranking plots of random forest for predicting frailty risk. ASCVD: atherosclerotic cardiovascular disease; BUN: blood urea nitrogen; CKD: chronic kidney disease; COPD: chronic obstructive pulmonary disease; PIR: poverty-to-income ratio; SHAP: Shapley additive explanations.

**Figure 8:**
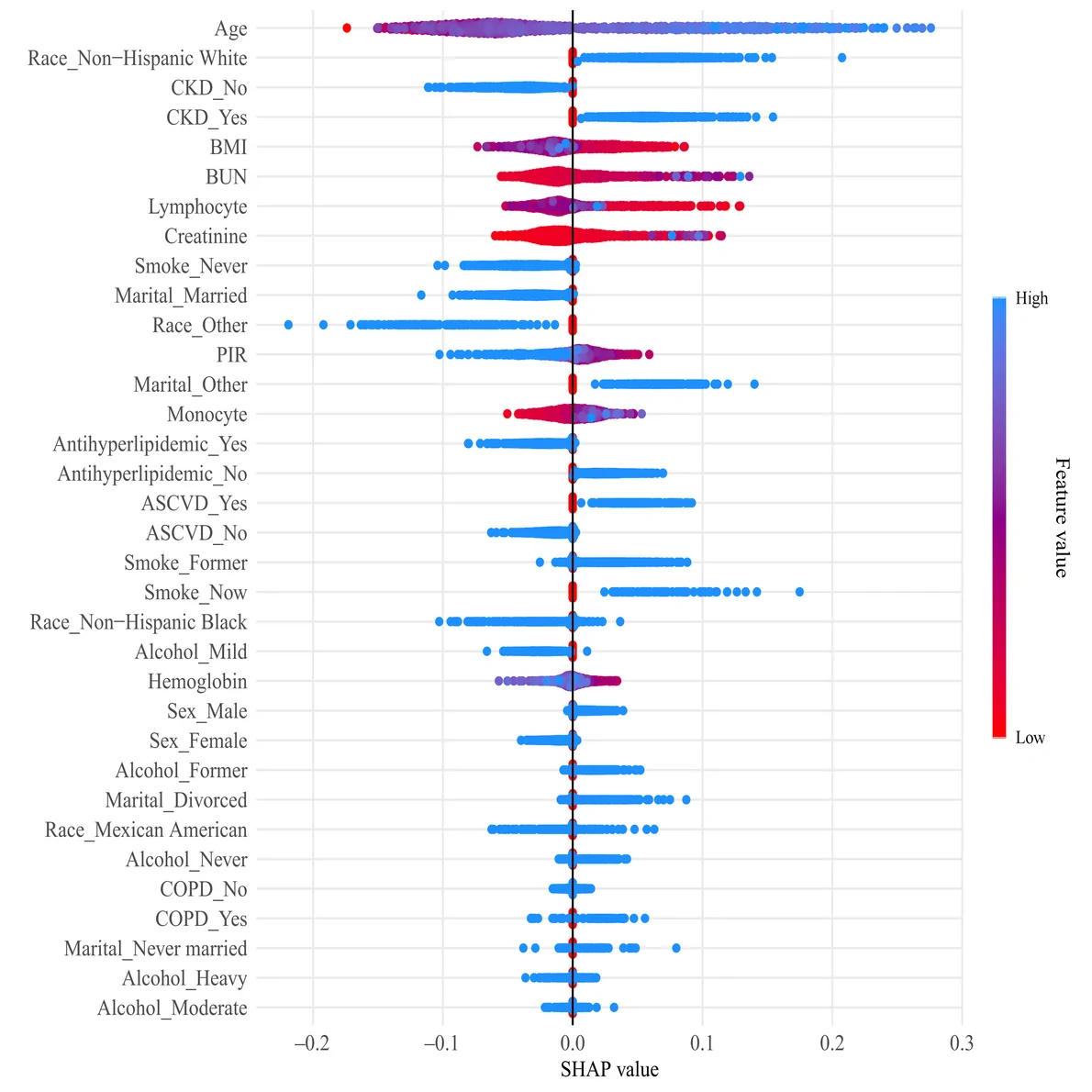
Summary plots of random forest for predicting frailty risk. ASCVD: atherosclerotic cardiovascular disease; BUN: blood urea nitrogen; CKD: chronic kidney disease; COPD: chronic obstructive pulmonary disease; PIR: poverty-to-income ratio; SHAP: Shapley additive explanations.

Figure 9 shows the SHAP dependence plots for continuous and categorical variables in the RF model. Figures 9 and 10 show that non-Hispanic White individuals and patients with CKD exhibited higher SHAP values, indicating elevated all-cause mortality risk in patients with T2DM and hypertension. Figures 11 and 12 show that as BMI increased, SHAP values initially decreased, but after exceeding a BMI threshold, SHAP values increased with further BMI elevation. Age also demonstrated a curvilinear relationship with all-cause mortality risk, with a cutoff value of 60 years; when age exceeded 60 years, patient all-cause mortality risk increased significantly. Similarly, BUN had a critical value of 20 mg/dL; when BUN was below 20 mg/dL, patient all-cause mortality risk decreased significantly, whereas when BUN exceeded 20 mg/dL, mortality risk increased significantly. Therefore, targeted feature management based on cutoff values derived from partial dependence plots may help reduce all-cause mortality risk in patients with T2DM and hypertension.

**Figure 9:**
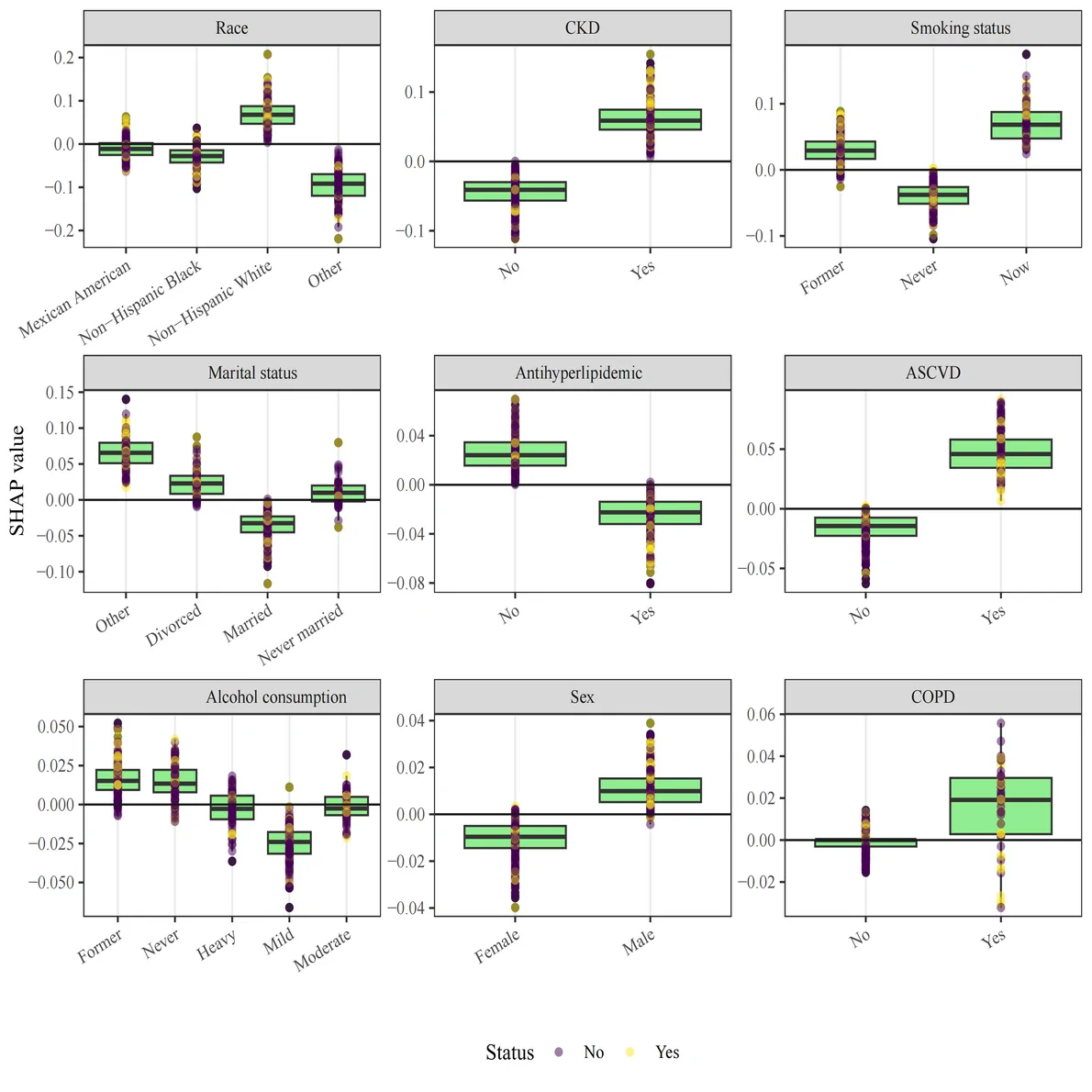
Shapley additive explanations (SHAP) dependency plots for continuous and categorical variables in the random forest model: partial dependence plot for some categorical variables. ASCVD: atherosclerotic cardiovascular disease; BUN: blood urea nitrogen; CKD: chronic kidney disease; COPD: chronic obstructive pulmonary disease; PIR: poverty-to-income ratio.

**Figure 10:**
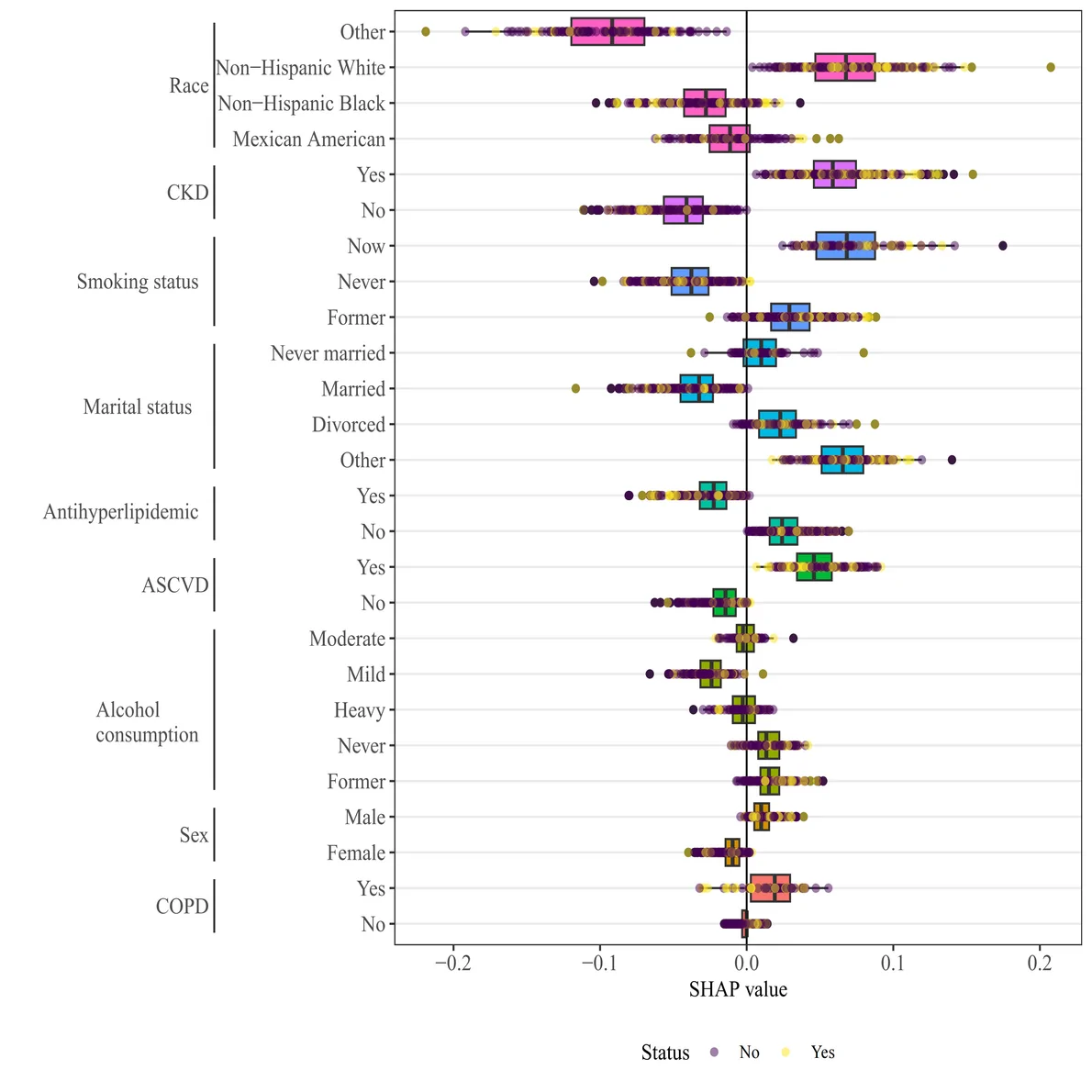
Shapley additive explanations (SHAP) dependency plots for continuous and categorical variables in the random forest model: partial dependence plot for some categorical variables. ASCVD: atherosclerotic cardiovascular disease; BUN: blood urea nitrogen; CKD: chronic kidney disease; COPD: chronic obstructive pulmonary disease; PIR: poverty-to-income ratio.

**Figure 11:**
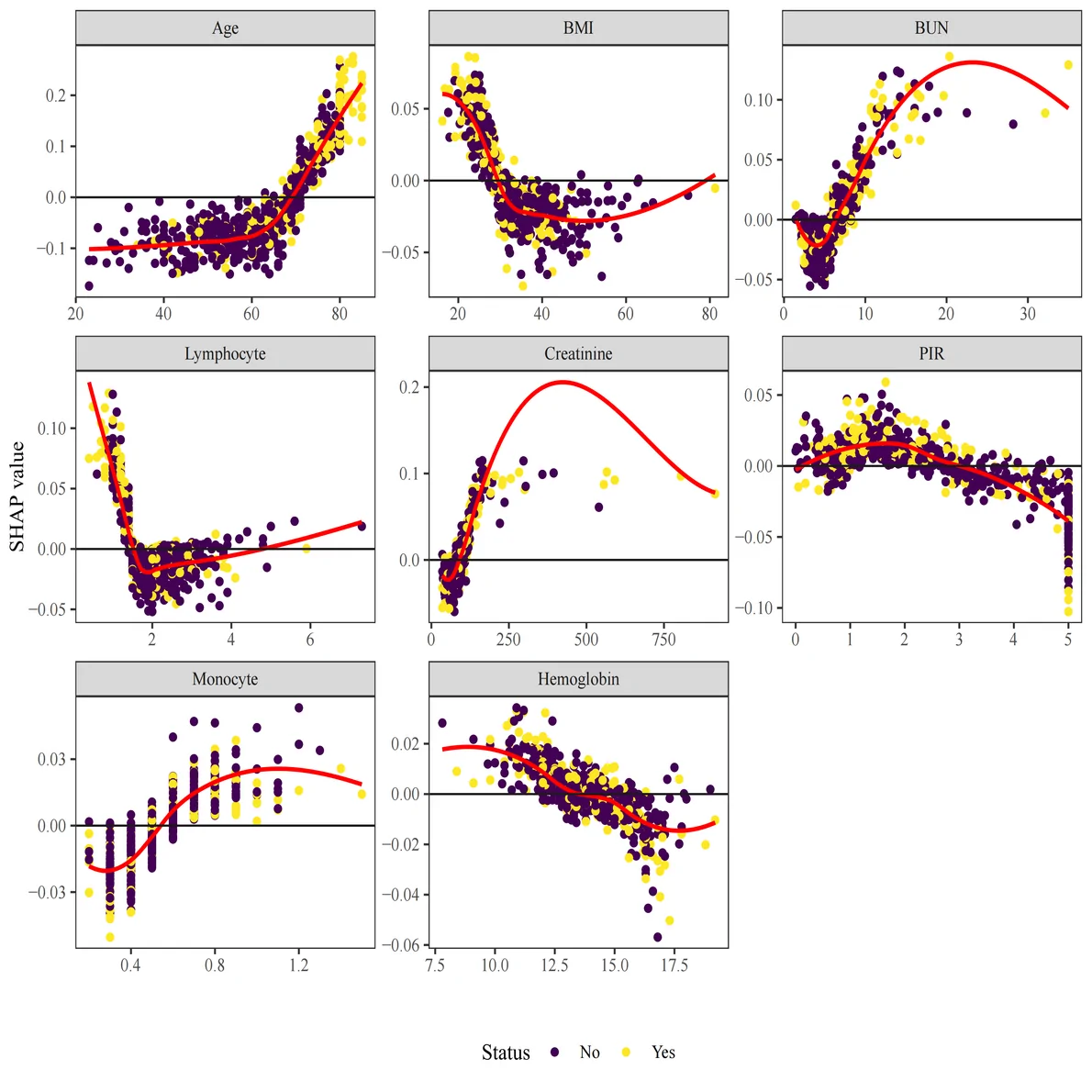
Shapley additive explanations (SHAP) dependency plots for continuous and categorical variables in the random forest model: partial dependence plot for some continuous variables. ASCVD: atherosclerotic cardiovascular disease; BUN: blood urea nitrogen; CKD: chronic kidney disease; COPD: chronic obstructive pulmonary disease; PIR: poverty-to-income ratio.

**Figure 12:**
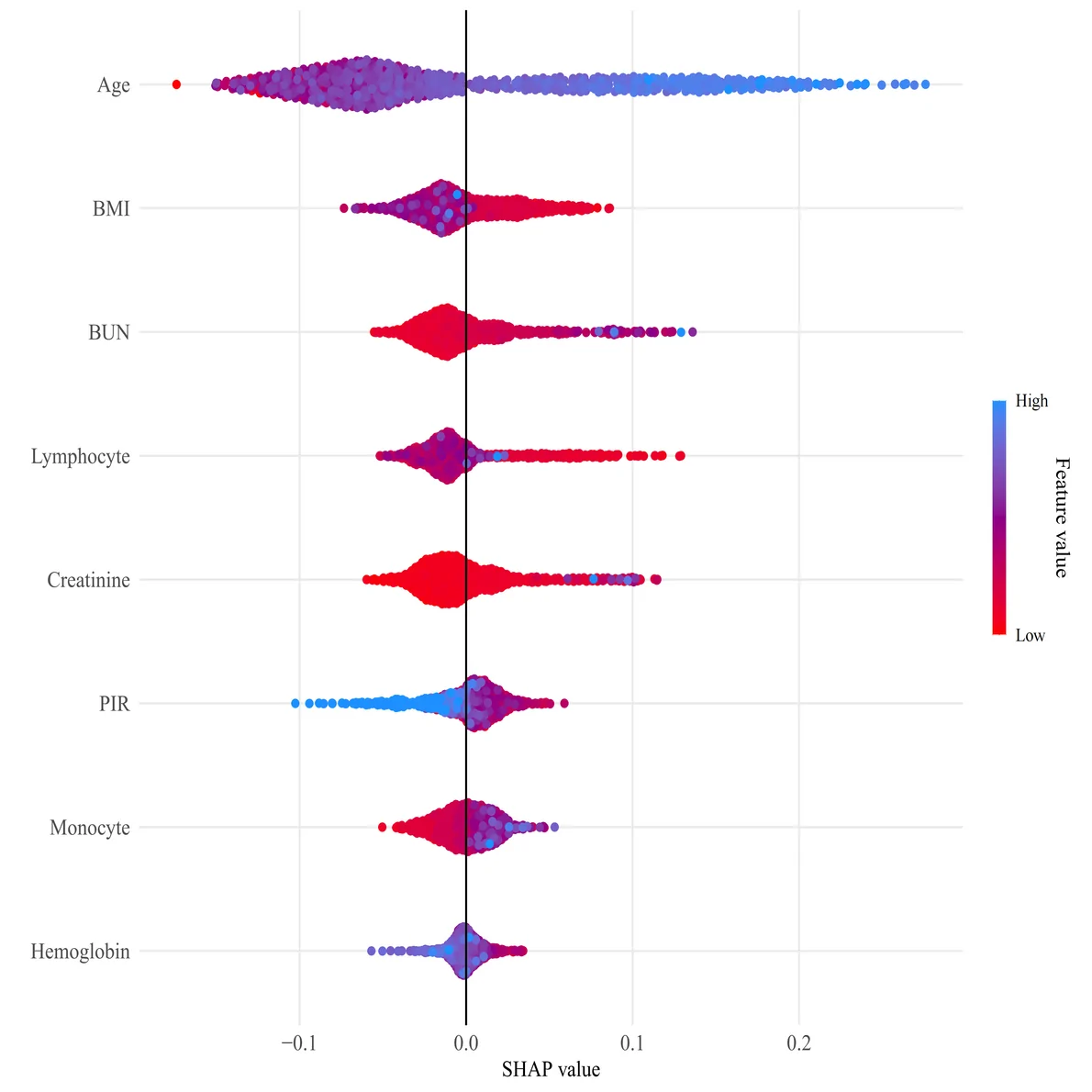
Shapley additive explanations (SHAP) dependency plots for continuous and categorical variables in the random forest model: partial dependence plot for some continuous variables. ASCVD: atherosclerotic cardiovascular disease; BUN: blood urea nitrogen; CKD: chronic kidney disease; COPD: chronic obstructive pulmonary disease; PIR: poverty-to-income ratio.

The waterfall plot (Figure 13A) illustrates a specific prediction for all-cause mortality risk in a patient with T2DM and hypertension: a non-Hispanic White patient aged 80 years with CKD whose SHAP value was elevated (0.732). In reality, this patient with T2DM and hypertension experienced all-cause death. Figure 13B shows the prediction for another patient with T2DM and hypertension who did not die: a non-Hispanic Black patient aged 60 years without CKD whose SHAP value was reduced (0.0556), with the prediction of no all-cause death occurring, consistent with the actual outcome. Force plots (Figures 13C and 13D) visually depict the SHAP values of each feature, determining the final prediction. Yellow and purple bars represent risk and protective factors, respectively, with longer bars indicating greater impact. These visualizations provide nuanced understanding of individualized risk factors, offering valuable insights for the practical application of prediction models in clinical settings.

**Figure 13:**
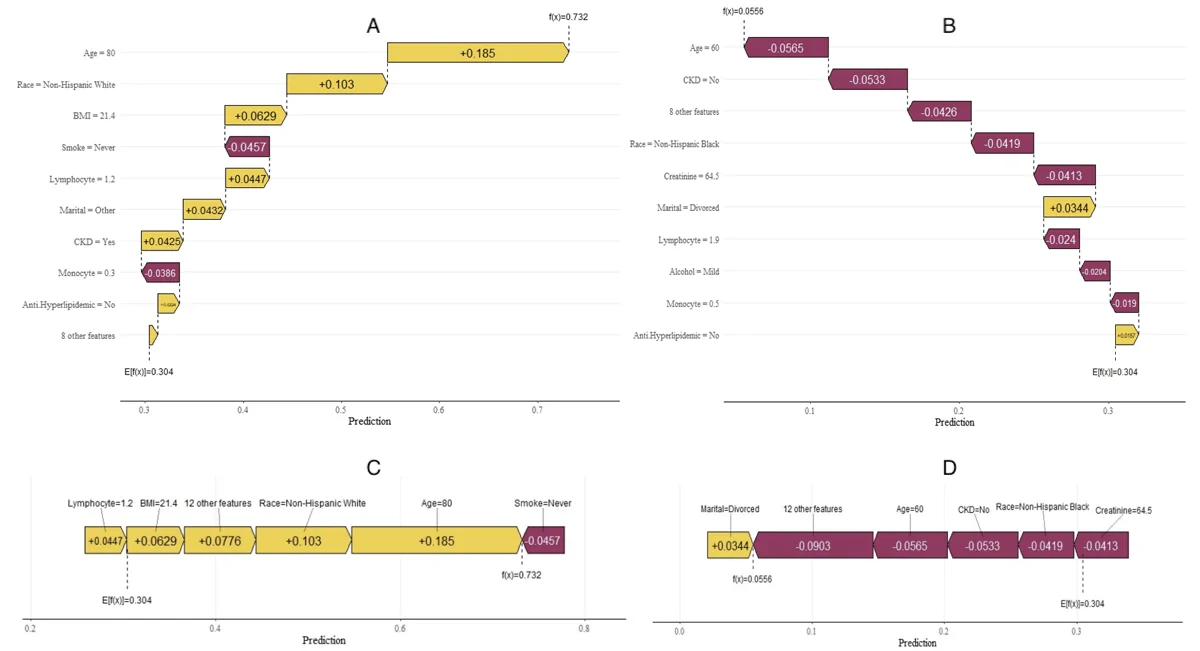
A and B: waterfall plots showing individual predictions for 2 patients with type 2 diabetes mellitus combined with hypertension. C and D: force plots visually depicting the Shapley additive explanations values of each feature, determining the final prediction. CKD: chronic kidney disease.

## Discussion

### Principal Findings

This study used LASSO regression of national cohort data to screen 17 key features from candidate variables and systematically compared 5 ML algorithms: LR, LightGBM, XGBoost, RF, and DT. Ultimately, the RF model demonstrated optimal performance in predicting all-cause mortality risk in patients with T2DM and hypertension. Through SHAP interpretability analysis, this study identified age as the most important predictor, with race, CKD, BMI, and BUN also showing significant influence and exhibiting distinct nonlinear relationship patterns such as critical threshold effects at the age of 60 years and BUN of 20 mg/dL. Individualized case analyses further validated the model’s clinical interpretability and predictive accuracy. These findings demonstrate both similarities and significant innovation and differences compared to previous studies in terms of methodological framework and clinical application value.

The LASSO regression feature selection strategy used in this study aligns with mainstream approaches for high-dimensional clinical data modeling in recent years. Research in statistical learning theory has emphasized the advantages of LASSO in handling high-dimensional data, particularly its ability to automatically perform variable selection while controlling overfitting [22]. Our approach of determining the optimal λ value through 10-fold cross-validation is entirely consistent with best practices recommended by Badillo et al [23] in their *Introduction to Statistical Learning*. Regarding feature selection results, the 17 variables identified in this study encompass multiple dimensions, including demographic characteristics, lifestyle factors, comorbidities, and laboratory indicators. This comprehensive feature composition is highly similar to variable selection patterns in previous cardiovascular risk prediction studies [24,25]. The multi-algorithm comparison research design also reflects the standardization trend of ML applications in the medical field. Large-scale algorithm comparison studies have indicated that no single algorithm can perform optimally across all datasets, necessitating systematic comparison to select the most appropriate method for specific problems.

In terms of model evaluation, the multidimensional assessment framework used in this study (AUC, accuracy, precision, recall, *F*_1_-score, calibration curves, and decision curve analysis) is consistent with international gold standards for prediction model research. Collins et al [26] clearly emphasized in the TRIPOD (Transparent Reporting of a Multivariable Prediction Model for Individual Prognosis or Diagnosis) statement that prediction model studies should report multiple evaluation dimensions, including discrimination ability, calibration, and clinical utility. Van Calster et al [27] further noted that the importance of calibration assessment in clinical prediction models is often underestimated, whereas our study validated model calibration performance through calibration curves. The decision curve analysis method developed by Vickers and Elkin [28] was used in this study to assess the clinical net benefit of the model, which has become a standard component of modern prediction model research.

However, this study differs from some previous studies in algorithm selection results. Multiple studies have shown that XGBoost and LightGBM typically perform excellently in medical prediction tasks [29]. The LightGBM model developed by Zhang et al [30] demonstrated dual advantages of speed and accuracy when processing large-scale data. Nevertheless, our study found that the RF model performed best, which aligns with the robustness and generalization capabilities emphasized in the original RF research [31]. These differences may reflect the impact of different data characteristics, sample sizes, and feature engineering strategies on algorithm performance.

Our study used SHAP for model interpretation, representing a cutting-edge trend in explainable AI applications in the medical field. Compared to traditional feature importance ranking, SHAP provides quantitative analysis of each feature’s contribution to specific prediction outcomes, which holds significant value in clinical decision support. However, it is crucial to emphasize that SHAP values reflect associational patterns within the observed data rather than establishing causal mechanisms, and our interpretations should be considered within this fundamental limitation. The nonlinear relationship patterns discovered through SHAP dependence plots in this study require careful interpretation within the context of potential confounding factors and structural determinants of health. Regarding the relationship between age and mortality risk, while Rao Kondapally Seshasai et al [32] found a continuous linear relationship between age and cardiovascular risk in a large-scale meta-analysis, the 60-year threshold effect identified in our analysis may reflect complex interactions among age-related physiological changes, health care use patterns, and treatment response characteristics specific to the T2DM population with hypertension rather than a direct causal threshold. The apparent nonlinear relationship between BMI and mortality risk, while partially consistent with previous “obesity paradox” research findings [33], may be influenced by unmeasured confounders, including health care access patterns, treatment intensity variations, and survival bias effects that are not captured in our cross-sectional analysis.

The racial differences observed in our SHAP analysis warrant particularly cautious interpretation as these patterns likely reflect complex structural and social determinants of health rather than intrinsic biological differences. While Spanakis and Golden [34] have documented the impact of racial factors on diabetes complications, the higher mortality risk associated with non-Hispanic White classification in our model may be confounded by multiple factors, including health care access disparities, socioeconomic differences, environmental exposures, historical health care inequities, and differential disease presentation patterns that are not adequately captured by the clinical variables in our dataset. These findings should be interpreted as highlighting potential health care disparities that require further investigation through studies designed to examine social determinants of health and structural factors contributing to differential outcomes. Similarly, while SHAP analysis identified CKD as the third most important predictor—a finding whose prominence may exceed expectations from previous studies—this association should be understood within the context of health care access, disease management quality, and potential surveillance bias. Although Go et al [35] emphasized the close association between CKD and cardiovascular risk, the relative importance of CKD in our model may reflect not only direct pathophysiological mechanisms but also differences in clinical monitoring, treatment intensity, and care coordination that influence mortality risk in patients with T2DM and hypertension. These interpretational limitations underscore the importance of viewing our SHAP findings as hypothesis-generating observations that identify potentially important associations warranting further investigation through prospective studies designed to examine causal pathways and address the influence of structural factors on health outcomes in diverse populations.

The discovery of BUN as an important predictor is also noteworthy. Traditionally, serum creatinine and estimated glomerular filtration rate have been considered better indicators of kidney function, but this study found that BUN has unique predictive value, echoing the research by Schrier [36] on the role of BUN in cardiorenal syndrome. The threshold effect of BUN at 20 mmol/L may reflect a critical point for the impact of kidney function impairment on prognosis in patients with T2DM and hypertension, providing an important reference for clinical monitoring. The individualized risk interpretation provided through waterfall plots and force plots in this study represents specific applications of precision medicine in clinical practice. This visualization method enables clinicians to understand the specific risk composition of each patient, providing support for individualized treatment decisions. Case analyses showed that a non-Hispanic White patient with CKD aged 80 years had high mortality risk (SHAP value=0.732), whereas a non-Hispanic Black patient without CKD aged 60 years had relatively low risk (SHAP value=0.0556). This individualized analytical capability transcends the population statistical characteristics of traditional risk scores, providing more refined risk stratification tools for clinical practice.

The successful translation of our RF model into clinical practice requires consideration of integration with existing health care infrastructure. The model’s architecture is well suited for digital implementation as it uses 17 routinely collected clinical variables (age, race, CKD, BMI, BUN, etc) that are standard components of electronic health record systems, requiring no additional data collection beyond routine clinical practice. For practical deployment, the model could be implemented as a web-based clinical calculator, integrated directly into electronic health record systems, or developed as a mobile app for point-of-care use. The SHAP-based interpretability framework provides transparent explanations for individual predictions through waterfall plots and force plots, which is essential for clinician acceptance and regulatory compliance in health care AI applications. This interpretability allows clinicians to understand exactly which factors drive risk assessment for each patient, supporting informed clinical decision-making. Implementation should follow a phased approach: phase 1 involving pilot-testing in select clinical sites to validate real-world performance and phase 2 incorporating automated performance monitoring and model maintenance protocols. The model’s strong calibration and clinical utility, demonstrated through decision curve analysis, suggest its potential value for risk stratification in patients with T2DM and hypertension, enabling more targeted interventions and resource allocation in clinical practice.

While our methodological framework incorporates established ML techniques, our study provides several distinct contributions that differentiate it from the standard prediction model pipeline commonly used in medical AI research.

### Methodological Innovations

Beyond the conventional approach of simply comparing algorithm performance metrics, our study integrated 3 key innovations:

- Disease-specific feature engineering—our LASSO-based feature selection specifically accounts for the pathophysiological interactions unique to concurrent T2DM and hypertension rather than treating these as independent conditions.
- Interpretability-guided model selection—unlike studies that select models based solely on AUC performance, our selection of RF was informed by both predictive accuracy and interpretability requirements for clinical decision-making.
- Threshold-based clinical insights—our SHAP analysis goes beyond traditional feature importance rankings to identify specific clinical thresholds (age of 60 years and BUN of 20 mg/dL) that can directly guide clinical management decisions.

### Clinical Application Innovation

Our approach differs significantly from standard ML pipelines by prioritizing clinical interpretability alongside predictive performance. The individualized risk visualization through waterfall plots and force plots provides patient-specific explanations that transcend population-level statistics, enabling personalized clinical decision-making. This represents a departure from “black-box” prediction models toward explainable AI that can support clinical reasoning.

### Population Health Innovation

Our study advances beyond conventional risk prediction by explicitly addressing health equity considerations. Rather than simply including race as a predictor variable, we provide careful interpretation of racial differences as reflections of structural determinants rather than intrinsic biological factors. This approach represents an important advancement toward developing AI tools that acknowledge and potentially help address health disparities. However, we acknowledge that these innovations, while meaningful, represent incremental rather than revolutionary advances in the field. Our contribution lies in the systematic integration of these approaches within a rigorously designed study using nationally representative data rather than breakthrough algorithmic development.

### Strengths and Limitations

This study has several important limitations that must be considered when interpreting our findings and their clinical applicability. First and most significantly, the lack of independent external validation represents a critical limitation that substantially restricts confidence in the model’s generalizability to other populations and health care settings. Our model was developed and validated using data exclusively from the US NHANES database, with internal validation through a 7:3 train-test split. While this approach follows appropriate methodological standards for initial model development, it cannot adequately assess the model’s performance when applied to populations outside the United States or in different health care systems. Second, our retrospective design relies on cross-sectional data, posing risks of information bias and lacking dynamic information about disease progression, treatment response, and changes in clinical status over time. Third, although multiple clinical indicators were included, key variables such as blood pressure control status, specific medication regimens, medication adherence, disease duration, and treatment intensity were missing from our analysis, which may introduce residual confounding and limit the model’s predictive accuracy. Fourth, the NHANES database, while nationally representative of the US population, may not capture the full spectrum of clinical phenotypes, treatment approaches, and health care access patterns that exist in diverse clinical settings. Finally, the interpretability of the RF model, while enhanced through SHAP analysis, remains limited compared to traditional statistical approaches, and the cross-sectional nature of our data cannot reflect dynamic clinical changes that occur during longitudinal patient management.

Overall, this study successfully developed and validated an all-cause mortality risk prediction model for patients with T2DM and hypertension based on large-scale NHANES cohort data. Through systematic comparison of 5 ML algorithms, the RF model significantly outperformed traditional LR and other algorithms. Age, race, CKD, BMI, and BUN were identified as key predictors, and the model demonstrated good calibration and clinical utility. This prediction tool provides scientific evidence and practical instruments for risk stratification, clinical decision-making, and individualized management of this high-risk population of patients with T2DM and hypertension.

## Appendix

Multimedia Appendix 1 Optimal hyperparameters of 5 machine learning models derived from 10-fold cross-validation and grid search, decision curve analysis evaluating net clinical benefit for these models in the training and test datasets, and calibration curves assessing the agreement between predicted probabilities and observed event rates across the training and test datasets.

## Abbreviations

ASCVD: atherosclerotic cardiovascular disease
AUC: area under the curve
BUN: blood urea nitrogen
CKD: chronic kidney disease
COPD: chronic obstructive pulmonary disease
DT: decision tree
LASSO: least absolute shrinkage and selection operator
LightGBM: light gradient boosting machine
LR: logistic regression
ML: machine learning
NHANES: National Health and Nutrition Examination Survey
PIR: poverty-to-income ratio
RF: random forest
SHAP: Shapley additive explanations
T2DM: type 2 diabetes mellitus
TRIPOD: Transparent Reporting of a Multivariable Prediction Model for Individual Prognosis or Diagnosis
XGBoost: extreme gradient boosting

## Data Availability

All relevant data are included within the manuscript and its supplementary data or in the publicly available National Health and Nutrition Examination Survey database [37].
