# Supplementary material for: Development and Validation of a Machine Learning–Based Model for Predicting All-Cause Mortality Risk in Patients With Type 2 Diabetes Mellitus Combined With Hypertension: National Cohort Study
Source: JMIR Med Inform. 2026 Sep 22;14:e85557. doi: 10.2196/85557 (PMC13643261; doi:10.2196/85557)
Supplement: Supplementary file 1 [file medinform_v14i1e85557_app1.doc]

**Development and Validation of a Machine Learning-Based Model for Predicting All-Cause Mortality Risk in Patients with Type 2 diabetes mellitus combined with hypertension: A National Cohort Study**

Wenlong Ding^1,+^,Lei Fang^2,+^,Cunming Fang^1,*^

^1^Department of Cardiology, Xuancheng Hospital Affiliated to Wannan Medical College (Xuancheng People 's Hospital), Xuancheng Anhui 242000, China;

^2^Department of Geriatrics Center,Tongling People's Hospital, Tongling, Anhui,244000,China;

***Corresponding Author:**

Cunming Fang,Department of Cardiology, Xuancheng Hospital Affiliated to Wannan Medical College (Xuancheng People 's Hospital), Xuancheng Anhui 242000, China;E-mail:fang19920629@outlook.com

^+^:These authors contributed equally and are regarded as co-first authors.

**Supplementary Table 1.Optimal hyper-parameter settings for five models based on 10-fold cross-validation and grid search strategy**

| **Methods** | **Optimal hyper-parameters** |
| --- | --- |
| **RF** | 'mtry':3,'trees':414,'min_n':48 |
| **XgBoost** | 'mtry':5,'min_n':19,'tree_depth':1,'learn_rate':0.0275,'loss_reduction':0.159,'sample_size':0.907 |
| **LR** | 'C': 10, 'solver': 'liblinear' |
| **LightGBM** | 'mtry':6,'trees':447,'min_n':10,'tree_depth':1,'learn_rate':0.0314,'loss_reduction':0.0588 |
| **DT** | 'cost_complexity':0.000354,'tree_depth':4,'min_n':6 |

**(A)	(B)**

**Supplementary Figure 1.Decision Curve Analysis of Machine Learning Models;(A)Training set(B)Validation Set;DT:Decision Tree,XGBoost:Extreme Gradient Boosting,LR:Logistic Regression,RF:Random forest,LightGBM:Light Gradient Boosting Machine.**

**(A)	(B)**

**Supplementary Figure 2.Calibration Curve of Machine Learning Models;(A)Training set(B)Validation Set;DT:Decision Tree,XGBoost:Extreme Gradient Boosting,LR:Logistic Regression,RF:Random forest,LightGBM:Light Gradient Boosting Machine.**
